# JPPRED: Prediction of Types of J-Proteins from Imbalanced Data Using an Ensemble Learning Method

**DOI:** 10.1155/2015/705156

**Published:** 2015-10-26

**Authors:** Lina Zhang, Chengjin Zhang, Rui Gao, Runtao Yang

**Affiliations:** ^1^School of Control Science and Engineering, Shandong University, Jinan 250061, China; ^2^School of Mechanical, Electrical and Information Engineering, Shandong University, Weihai 264209, China

## Abstract

Different types of J-proteins perform distinct functions in chaperone processes and diseases development. Accurate identification of types of J-proteins will provide significant clues to reveal the mechanism of J-proteins and contribute to developing drugs for diseases. In this study, an ensemble predictor called JPPRED for J-protein prediction is proposed with hybrid features, including split amino acid composition (SAAC), pseudo amino acid composition (PseAAC), and position specific scoring matrix (PSSM). To deal with the imbalanced benchmark dataset, the synthetic minority oversampling technique (SMOTE) and undersampling technique are applied. The average sensitivity of JPPRED based on above-mentioned individual feature spaces lies in the range of 0.744–0.851, indicating the discriminative power of these features. In addition, JPPRED yields the highest average sensitivity of 0.875 using the hybrid feature spaces of SAAC, PseAAC, and PSSM. Compared to individual base classifiers, JPPRED obtains more balanced and better performance for each type of J-proteins. To evaluate the prediction performance objectively, JPPRED is compared with previous study. Encouragingly, JPPRED obtains balanced performance for each type of J-proteins, which is significantly superior to that of the existing method. It is anticipated that JPPRED can be a potential candidate for J-protein prediction.

## 1. Introduction

J-proteins, a prototypical molecular chaperone family, were originally identified in* Escherichia coli* by Georgopoulos et al. [1] and found ubiquitously in cells from prokaryotes to eukaryotes [2]. J-proteins, also called heat shock protein 40s (HSP40s), act as obligate cochaperone partners of the Hsp70 chaperone to participate in a variety of cellular processes by interacting with HSP70s through the specific J-domain and activating the chaperone activity of HSP70s [3]. The J-proteins combined with HSP70s are probably recognized as self-antigens which may be tumor makers for cancers [4]. They can be regarded as prediction standard to diagnose cancers early [5] and critical in congenital and adaptive immunity [6]. In addition, J-proteins play significant roles in response to cellular stress, including refolding of proteins damaged by harmful stresses [7] and degradation of misfolded proteins [8].

J-proteins have 4 distinct types based on the composition of domains, including a signature J-domain with its conserved His, Pro, and Asp (HPD) motif, a Gly/Phe-rich region, a CXXCXGXG zinc-finger domain, and a less conserved C-terminal substrate-binding domain [9]. Type I J-proteins are characterized by all the 4 regions. Type II J-proteins lack the zinc-finger domain. Type III J-proteins only contain the J-domain. Type IV J-proteins have been recently described and classified as “J-like proteins,” exhibiting variations in the HPD motif of J-domain [2]. The structures of peptide-binding sites for 4 types of J-proteins might be distinct from each other, which can lead to remarkable differences in their chaperone functions [9]. Types I J-proteins can suppress protein aggregation and facilitate the refolding of damaged proteins [10]. Types II J-proteins are implicated in protein folding processes and can help translation initiation and protein degradation [11]. Type III J-proteins are more structurally and functionally divergent and involved in protein translocation [12]. Type IV J-proteins seem to interact with HSP70s in a different manner to exert their functions [13].

Different types of J-proteins have distinct roles in the development of diseases. Type I J-proteins may provide significant clues to develop plasmodium-specific J-proteins inhibitors against malaria infection [13]. In addition, type I J-proteins are tumour promoting [15], while type II J-proteins may be largely regarded as tumor suppressors [16]. The type II J-proteins can also participate in promoting degradation of terminally misfolded cytosolic proteins [17], which may provide new ideas to treat or cure conformational diseases. The type IV J-proteins are a very promising group in terms of potential drug targets, as indicated in [13].

In view of the different functions in biological processes and organisms, accurate identification of types of J-proteins will be of benefit to gain novel insights into the mechanism of J-proteins and contribute to developing drugs to cure or alleviate some types of diseases. The explosive growth of protein sequences generated in the postgenomic age has made a large gap between the number of sequence-known and the number of structure-known proteins [18]. Therefore, it would be urgent to develop computational methods for rapidly and effectively identifying the types of J-proteins.

To the best of our knowledge, only one machine learning method has been proposed to identify the types of J-proteins. Feng et al. [14], using the tripeptide composition of reduced amino acid alphabet as the encoding scheme, presented a support vector machine based method to identify the types of J-proteins. This method has its own merits but achieves severely unbalanced performance for the 4 types of J-proteins, which may attribute to the following shortcomings. (1) This method used a single feature extraction strategy. Generally, multiple features can not only preserve enough discriminative information for protein attribute predictions, but also complement each other to enhance the performance and robustness of a predictor [19]. Thus, hybrid features have been increasingly used in recent studies for constructing classifiers [20, 21]. (2) The earlier work did not apply feature selection method to select the high discriminative features from the tripeptide composition of reduced amino acid alphabet, which would lead to dimension disaster and poor performance [22]. Feature selection is essential to remove the redundancy information or noise existing in the extracted features and decrease the models complexity in classification problems [23]. (3) The existing method was based on individual classifier, which could have its own inherent defects limiting the prediction performance [24]. In general, the ensemble classifier that integrates multiple basic classifiers of diverse learning policies can perform better than its component classifiers [25]. (4) The method did not deal with the serious class imbalance problem, which would lead to high prediction accuracy for the majority class but poor prediction accuracy for the minority class [26].

**Figure 1:**
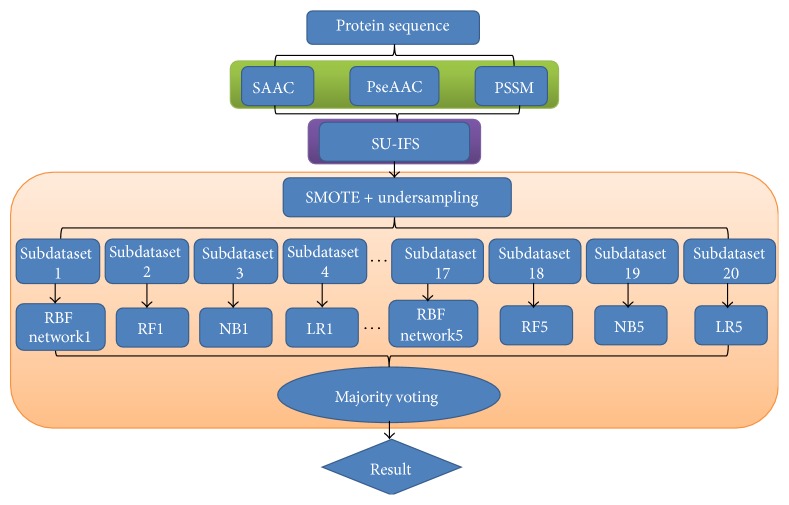
The computational framework of the proposed method. SAAC: split amino acid composition; PseAAC: pseudo amino acid composition; PSSM: position specific scoring matrix; SU: symmetric uncertainty; IFS: incremental feature selection; SMOTE: synthetic minority oversampling technique; RBF: radial basis function; RF: random forest; NB: naïve Bayes; LR: logistic regression.

In order to address the above-mentioned limitations and improve the performance for identifying the types of J-proteins, this study puts forward an ensemble method with hybrid features extracted from SAAC, PseAAC, and PSSM. The proposed method is implemented in the following steps. (1) The benchmark dataset is obtained from heat shock protein information resource (HSPIR). (2) Protein sequences are converted into a numerical feature vector based on SAAC, PseAAC, and PSSM. (3) SU-IFS is adopted to obtain the optimal feature set. (4) The SMOTE and undersampling technique are applied to deal with the imbalanced benchmark dataset. (5) The ensemble method is developed by integrating 20 subclassifiers trained by 20 subdatasets based on 10-fold cross validation. (6) The predicted class label is determined based on the majority voting strategy. To evaluate the prediction performance of the proposed ensemble predictor objectively, the present model is compared with [14]. The proposed method will be referred to as JPPRED (J-Protein PREDiction) in the rest of the paper. The computational framework of the proposed method is illustrated in Figure 1.

## 2. Materials and Methods

### 2.1. Data Collection

J-protein sequences are collected from HSPIR [27] at http://pdslab.biochem.iisc.ernet.in/hspir/hsp40.php, which contains 3901 sequences.

In order to obtain a reliable and high quality dataset, the following criteria are further performed. (1) Sequences which are fragments of other proteins are excluded because their information is redundant and not integrity. (2) Sequences containing nonstandard letters such as “B,” “X,” or “Z” are excluded because their meanings are ambiguous. (3) The sequence identity cutoff threshold is set as 40% to dislodge the redundant sequences using CD-HIT program [28]. After the above screening procedures, the final benchmark dataset consists of 1199 J-protein sequences, including 63 type I J-proteins, 55 type II J-proteins, 1061 type III J-proteins, and 20 type IV J-proteins (see Table  S1, in Supplementary Material available online at http://dx.doi.org/10.1155/2015/705156). Since the original benchmark dataset is processed by excluding the sequences which contain nonstandard letters, there are slight differences between the numbers of different types of J-proteins in our study and those in [14].

### 2.2. Feature Extraction

To develop an accurate prediction model for pattern recognition problems in bioinformatics, one of the key steps is to represent the protein sequences with appropriate descriptors that can truly reflect the intrinsic correlation with the target sequences to be predicted [29]. In general, an individual feature extraction strategy can only represent partial target's knowledge, which could limit the prediction performance. Multiple features can take full advantage of the supplementary information from different features to enhance the prediction accuracy. With this in mind, a diverse set of features extracted from SAAC, PseAAC, and PSSM are adopted in this study to encode protein sequences.

#### 2.2.1. Split Amino Acid Composition

Previous study has explored and compared the frequencies of 20 nature amino acids among the 4 types of J-proteins, which indicates that the frequencies of the 19 nature amino acids are remarkably different [14]. Therefore, it is reasonable to extract features from amino acid composition (AAC).

**Figure 2:**
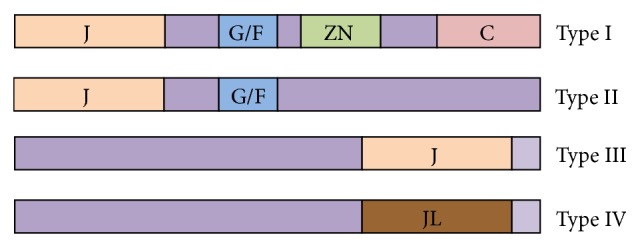
Structural classification of J-proteins (http://pdslab.biochem.iisc.ernet.in/hspir/hsp40.php).

In view of different domain organizations of 4 types of J-proteins as shown in Figure 2, three-part composition based on split amino acid composition (SAAC) is adopted in the study. Compared with the traditional amino acid composition (AAC), split amino acid composition (SAAC), the successor of AAC [30], takes the importance of the N-terminal and C-terminal into account and assigns adequate weight to the compositional bias [31], which is known to be present in the protein terminus [32]. Therefore, it has been widely applied in protein function predictions [33, 34] and achieves excellent results.

Based on SAAC, a given protein sequence is split into 3 parts including N-terminal, C-terminal, and the remaining center portion. The AAC of each part is calculated separately and merged together to obtain a 60-dimension feature vector. The discriminative power of the features based on different lengths of N or C-terminal varying from 15 to 25 is investigated.

#### 2.2.2. Pseudo Amino Acid Composition

To include the global or long-range sequence-order information, the concept of pseudo amino acid composition [35] was proposed. Since then, the PseAAC approach has rapidly penetrated into many areas of computational proteomics [36–39] and a long list of references cited in a review [29]. Thus, in this paper, we also use the concept of PseAAC to construct a correlation factor to describe the long-range sequence-order information.

Being the most intuitive features for protein biochemical reactions, physicochemical properties of amino acids have a deep influence on the diversity and specificity of protein structures and functions [40]. Features incorporating physicochemical properties can contain much valuable information for improving the performance of a predictor. It is really important to choose appropriate physiochemical properties for residue representation.

To extract features from physicochemical properties with PseAAC, we consider 12 important physiochemical properties, including hydrophobicity, hydrophilicity, average accessible surface area, average flexibility indices, net charge, side chain volume, polarity, heat capacity, isoelectric point, transfer free energy to surface, van der Waals, and side chain interaction parameter. For a given protein sequence with the length of *L*, PseAAC can be represented by discrete correlation factors

(1) F=f1,f2,…,fγT,γ<L,fγ=1L−γ∑i=1L−γpij−1L∑i=1Lpijpi+γj−1L∑i=1Lpij,

where *p*_*ij*_ is the *j*th physicochemical property value of the amino acid at the *i*th position. *γ* denotes the distance between one residue and its neighbor at a certain number of residues away, which is closely related with sequence order information and performs an important role in the predictive quality of a model. The discriminative power of the features based on different *γ* varying from 1 to 10 is investigated.

#### 2.2.3. Position Specific Scoring Matrix

Protein sequences have developed starting from a very finite number of ancestral species protein sequences, which evolves involving changes, insertions, and deletions of single or several residues [41]. Ultimately, two protein sequences may have a few distinct amino acid residues, but they may still share some structure similarities and the same function [42]. Therefore, evolutionary conservation can determine important biology functions [43]. Among the domain organizations of J-proteins, C-terminal domains are less conserved, and the other 3 domains are all conserved motifs [44].

Evolutionary conservations can be obtained by the position specific scoring matrix (PSSM), which has been proved to be highly effective in protein attribute predictions [20, 45]. Generated by PSI-BLAST [46], PSSM profile is composed of *L∗*20 elements for a given protein sequence with length of *L*, defined as

(2) $$\begin{array}{c} P_{\text{PSSM}}=\left[\begin{array}{cccccc} E_{1\to 1} & E_{1\to 2} & \cdots & E_{1\to j} & \cdots & E_{1\to 20}\\ E_{2\to 1} & E_{2\to 2} & \cdots & E_{2\to j} & \cdots & E_{2\to 20}\\ \vdots & \vdots & \cdots & \vdots & \cdots & \vdots \\ E_{i\to 1} & E_{i\to 2} & \cdots & E_{i\to j} & \cdots & E_{i\to 20}\\ \vdots & \vdots & \cdots & \vdots & \cdots & \vdots \\ E_{L\to 1} & E_{L\to 2} & \cdots & E_{L\to j} & \cdots & E_{L\to 20} \end{array}\right], \end{array}$$

where the values in the *i*th row are the probabilities of the *i*th residue in a given protein sequence mutating to 20 native amino acids. Previous study has illustrated that normalizing the PSSM can weaken noise and bias in the original elements to improve the prediction performance [47]. The PSSM is normalized using the following sigmoid function to scale each element to a range of 0 to 1:

(3) $$\begin{array}{c} f\left(\;x\;\right)=\frac{1}{1+e^{-x}}, \end{array}$$

where *x* is the original PSSM value.

Autocovariance (AC), depicting the average interactions between two residues, has been successfully adopted to grasp the local discriminative information [48]. To acquire more evolutionary and local sequence order information, AC is adopted to extract features from PSSM and defined as

(4) AC⁡λ=1L−λ∑i=1L−λEi→j−Ej¯Ei+λ→j−Ej¯,j=1,2,…,20;  λ=1,2,…,K,

where $\bar{E_{j}}$ is the average value along the *j*th column in the PSSM. *λ* is the distance between two considered amino acid residues, which is closely related to sequence order information and plays an important role in the performance of a predictor. Therefore, we evaluate the discriminative power of the features based on different *λ* varying from 1 to 5.

### 2.3. Feature Selection

After running the hybrid feature extraction methods, primary protein sequences are converted into numerical feature vectors with the same dimension. The prediction performance is largely based on discriminative features. However, the simple combination of features extracted from different methods may bring information redundancy and noise, which can cause dimension disaster and deteriorate the discriminative power of the classifiers [22].

Feature selection techniques are essential to pick out informative features and gain deeper insights into intrinsic properties of protein sequences, which can prevent overfitting, enhance the efficiency, and improve the prediction quality [49].

The optimal feature set can be achieved by examining the performance of all combinations of features. However, it has heavy computing burden. To economize computational resource, the symmetric uncertainty (SU) attribute evaluator combined with incremental features selection (IFS) is adopted in this study to obtain the optimal feature set.

#### 2.3.1. Symmetric Uncertainty

Symmetrical uncertainty (SU) [50], a normalized information theoretic measure, is employed to evaluate the relevancy of each feature with respect to the class based on entropy and conditional entropy values.

The SU of the feature variable *f*_*i*_ and the class variable *C* is measured by

(5) $$\begin{array}{c} \text{SU}\left(\;f_{i},C\;\right)=2\left[\;\frac{\text{IG}\left(f_{i}\;|\;C\right)}{H\left(f_{i}\right)+H\left(C\right)}\;\right]. \end{array}$$

Denote a set of values of *f*_*i*_ as {*f*_*i*_^1^, *f*_*i*_^2^,…, *f*_*i*_^*j*^,…, *f*_*i*_^*n*^}. Information entropy that measures the uncertainty of a feature variable *f*_*i*_ is calculated as

(6) $$\begin{array}{c} H\left(\;f_{i}\;\right)=-\overset{n}{\underset{j=1}{\sum }}P\left(\;f_{i}^{j}\;\right)\log_{2}\left(\;P\left(\;f_{i}^{j}\;\right)\;\right), \end{array}$$

where *P*(*f*_*i*_^*j*^) represents the prior probability of *f*_*i*_^*j*^.

The information entropy of class variable *C* is formulated by

(7) $$\begin{array}{c} H\left(\;C\;\right)=-\underset{i}{\sum }P\left(\;c_{i}\;\right)\log_{2}\left(\;P\left(\;c_{i}\;\right)\;\right), \end{array}$$

where *P*(*c*_*i*_) represents the prior probability of *c*_*i*_ and *c*_*i*_ denotes one of values of class variable *C*.

The entropy *H*(*C*∣*f*_*i*_) of *C* after observing *f*_*i*_ is calculated as

(8) HC ∣ fi=−∑jPfij∑iPci ∣ fijlog2⁡Pci ∣ fij,

where *P*(*c*_*i*_∣*f*_*i*_^*j*^) is the posterior probability of *c*_*i*_ given the value *f*_*i*_^*j*^ of *f*_*i*_.

Information gain IG(*f*_*i*_∣*C*) that represents the amount by which the entropy of *f*_*i*_ decreases provided by class variable *C* is defined as

(9) $$\begin{array}{c} \text{IG}\left(\;f_{i}\;|\;C\;\right)=H\left(\;C\;\right)-H\left(\;C\;|\;f_{i}\;\right). \end{array}$$

SU normalizes the values of information gain within range [0,1] [51]. The feature which has high value of SU is more relevant to the class label. According to values of SU, the ranked feature list can be acquired. The smaller the index is, the more relevant the feature is. The WEKA (Waikato Environment for Knowledge Analysis) software package is used for the feature selection algorithm SU, where default parameters are employed. The software package can be downloaded at http://www.cs.waikato.ac.nz/ml/weka/downloading.html.

#### 2.3.2. Incremental Feature Selection

Based on the ranked feature list according to the relevance to the class evaluated by SU, the incremental feature selection (IFS), one of the well-known searching strategies of feature selection, is employed to determine the optimal features.

The IFS procedure starts with an empty subset and adds features in the ranked feature list one by one from higher to lower rank into the feature subset [49]. When a new feature is added, a new feature subset is generated. The *i*th feature subset can be formulated as

(10) $$\begin{array}{c} F_{i}=\left\{\;f_{1},f_{2},\ldots ,f_{i}\;\right\}\;\left(1\leq i\leq N\right). \end{array}$$

For each feature subset *F*_*i*_, an ensemble predictor is constructed and tested using 10-fold cross validation test. The feature subset that yields the best prediction performance and has lower dimension is determined as the optimal one.

### 2.4. Ensemble Learning Method

Single classifier has its own shortcomings and could not always perform well on all datasets [52]. Ensemble learning emerges as the promising measure to overcome this problem. A well-defined ensemble of multiple classifiers has been proved to achieve better prediction performance than its component individual classifiers, which has been increasingly and widely applied in protein attribute prediction problems [53, 54].

The classification performance of an ensemble classifier is based on diversity and individual accuracy of its individual component [55]. Diversity represents the multiple classifiers that have diverse learning strategies while individual accuracy means the explored classifiers all have excellent individual prediction performance [56]. Identifying types of J-proteins is a multiple classification problem. The one-versus-rest strategy is adopted in this study.

**Figure 3:**
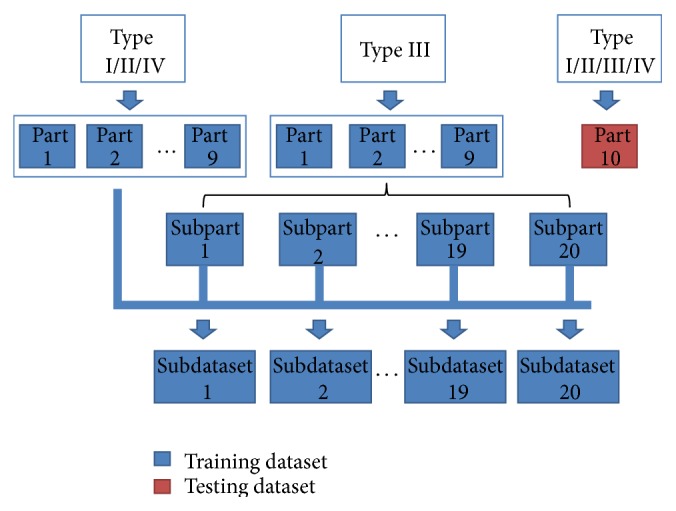
The generation process of 20 subdatasets in the first round of 10-fold cross validation.

As indicated in Section 2.1, the benchmark dataset contains nearly equal number of type I and type II J-proteins. The type IV J-proteins account for the vast majority of the benchmark dataset. On the contrary, there are few type IV J-proteins in the benchmark dataset. To deal with the imbalanced benchmark dataset, the number of type IV J-proteins is expanded to 60 based on SMOTE [57]. Then, an ensemble learning method based on the undersampling technique is utilized for identifying different types of J-proteins. Using the first round of 10-fold cross validation as an example, the specific procedures of the undersampling technique are as shown in Figure 3.

Based on the theory of 10-fold cross validation, type I, type II, type III, and type IV J-proteins are randomly divided into 10 equally sized parts, respectively. In the first round, the testing dataset is composed of the tenth part from type I, type II, type III, and type IV J-proteins. The remaining J-proteins form the training dataset. Then, type III J-proteins from the training dataset are further processed by dividing them into 20 equally sized subparts. Type I, type II, and type IV J-proteins from the training dataset are combined with each subpart from type III J-proteins to construct 20 subdatasets. Ensemble classifiers, including radial basis function network, random forest, naïve Bayes, and logistic regression, are trained by these 20 subdatasets, respectively. The final class label is determined based on the majority voting strategy. This process is repeated 10 times to traverse every part of type I, type II, type III, and type IV J-proteins. If more than one class label obtains the same votes, a given protein is classified as the class label that has the nearest distance from the feature vector of the given protein.

### 2.5. Performance Measures

In statistical prediction, there are 3 cross validation methods to examine the performance of a predictor, including independent dataset test, subsampling test (e.g., 5-fold or 10-fold cross validation), and jackknife test [58]. Among these three methods, the jackknife test is deemed the most objective and rigorous one that can exclude the memory effects during the entire testing process and can always yield a unique result for a given benchmark dataset, as elucidated in [59] and demonstrated by Equation  50 of Chou and Shen [60]. Therefore, the jackknife test has been increasingly and widely adopted by investigators to test the power of various predictor [38, 61–64]. To reduce the computational complexity, we adopt the 10-fold cross validation test in this study. The benchmark dataset is randomly divided into 10 equally sized parts, where 9 parts are merged as one training set to develop a model and then the model is tested by the remaining part. This process is repeated 10 times to ensure every part as the testing set once. The ultimate result is the average of the 10 prediction results.

To assess the performance of the predictor intuitively, sensitivity (Sn), specificity (Sp), and accuracy (Acc) are employed, which are defined as

(11) Sni=TPiFNi+TPi,Spi=TNiFPi+TNi,Acc=∑i=14TPiN,

where TN, TP, FN, and FP stand for the number of true negative, true positive, false negative, and false positive, respectively. *i* represents the type of the target sample. *N* is the total number of the samples.

Due to the distinct numbers of types of J-proteins in the benchmark dataset, average Sn (AvgSn) is proposed to further test the predictive power more objectively, which is formulated as

(12) $$\begin{array}{c} \text{AvgSn}=\frac{1}{4}\overset{4}{\underset{i=1}{\sum }}\text{Sn}\left(\;i\;\right). \end{array}$$

## 3. Results and Discussions

### 3.1. Optimal Parameters for Individual Feature Spaces

To achieve the best characterization of protein sequences, we first evaluate the impact of key representative parameters on the prediction performance of individual feature spaces. JPPRED is constructed for each of individual feature spaces, including SAAC, PseAAC, and PSSM.

A good prediction system is usually expected to provide high sensitivity for every class lable. Therefore, AvgSn is introduced as the optimization objective to determine the corresponding optimal parameters, respectively, Lnc for SAAC, *λ* for PSSM, and *γ* for PseAAC.

**Figure 4:**
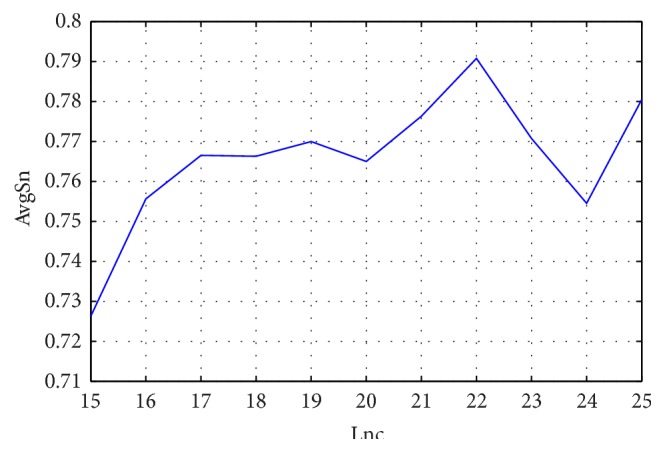
Performance of JPPRED using SAAC based features with different lengths of N- and C-terminals (Lnc).

Figure 4 gives the classification results using SAAC based features with different lengths of N- and C-terminals (Lnc). As Lnc increases, AvgSn almost monotonically increases in the initial phase. Afterwards, AvgSn is fluctuating with the increase of Lnc. JPPRED achieves the highest AvgSn of 0.791 when 22 amino acids on both the N- and the C-terminals are selected to extract features from SAAC. SAAC discriminates the types of J-proteins with an acceptable AvgSn because it considers the amino acid composition of the signal peptide on both the N- and the C-terminals. It reveals that the frequencies of 20 nature amino acids of N-terminal, C-terminal, and middle parts are remarkably different among different types of J-proteins, which is consistent with the results in [14]. Therefore, SAAC based features are reasonable to identify the types of J-proteins.

**Figure 5:**
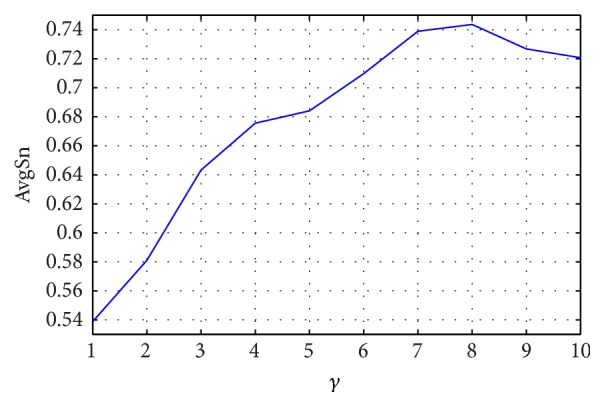
Performance of JPPRED using PseAAC based features with various *γ*.

The prediction performance of JPPRED using PseAAC based features with different *γ* varying from 1 to 10 is illustrated in Figure 5. From Figure 5, JPPRED achieves the best AvgSn of 0.744 at *γ* = 8. Based on physicochemical properties, PseAAC based features take into account the knowledge of sequence order, achieving a passable prediction performance. This is the first attempt to employ PseAAC to identify the types of J-proteins, which may help provide new annotations for the properties of different types of J-proteins.

**Figure 6:**
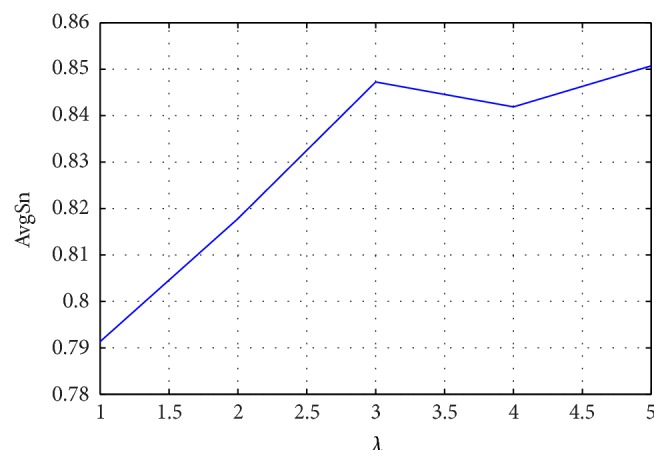
Performance of JPPRED using PSSM based features with various *λ*.

The parameter *λ* represents the distance between two amino acids in the sequence. AC along each column of PSSM represents the neighboring effect between amino acids and evolutionary information in a given protein sequence. Performance predictions of JPPRED using PSSM based features with different *λ* are shown in Figure 6. The highest AvgSn of 0.851 is obtained for *λ* = 5. PSSM based features take into the sequence order information consideration and also preserve the evolution information of the protein sequence. They yield the best prediction performance among the individual feature spaces. These results demonstrate that there is a big difference of evolution conservation among different types of J-proteins, which is in accordance with [44].

### 3.2. Performance Analysis of Ensemble Learning Method Using Different Feature Spaces

In order to explore the effectiveness of various feature spaces, the prediction results constructed by individual and hybrid feature spaces are listed in Table 1. Individual feature spaces identify the types of J-proteins with AvgSn ranging from 0.744 to 0.851, indicating that all the 3 individual feature spaces have acceptable discrimination power. PSSM based features discriminate the types of J-proteins with best performance among the 3 feature spaces with AvgSn of 0.851 and Acc of 0.808. Moreover, the PSSM information also has shortcomings. The generation of PSSM of a protein depends largely on the searching dataset. If no homologous sequence is found in the searching dataset, the PSSM cannot be obtained [49]. In the implementation process of our proposed method, when there is no homologous sequence of a given protein in search dataset, we assign a zero matrix to the PSSM of the protein. As a minority of sequences have no homologous sequences in the benchmark dataset, the overall prediction performance of JPPRED will not be affected. So PSSM is an appropriate feature extraction strategy here. It is worth mentioning that Acc of these individual feature spaces are relatively low, essentially due to the imbalance in the numbers of different types of J-proteins.

As shown in Table 1, the hybrid feature space of SAAC and PseAAC achieves better prediction performance compared to that of SAAC based features and that of PseAAC based features. The same result occurs in the hybrid feature space of PseAAC and PSSM. However, the hybrid feature space of SAAC and PSSM performs worse compared to the PSSM based features. This phenomenon may be due to the fact that SAAC introduces some redundancy features in the hybrid feature space of SAAC and PSSM. It should be noted that, compared to the hybrid space of SAAC and PSSM, the combination of PseAAC and PSSM can better enhance the prediction quality of JPPRED. Furthermore, JPPRED yields the highest AvgSn of 0.875 using the combination of SAAC and PseAAC in conjunction with PSSM based features, about 1.2% higher than that achieved by hybrid feature spaces of PSSM and PseAAC. Other performance measures have also indicated powerful discriminant ability of JPPRED using the hybrid feature spaces.

The obtained results reveal that different feature spaces include diverse types of information and contribute to the prediction accuracy differently. Any feature spaces that may show poor performance on certain protein attributes prediction cannot be declared as nondiscriminative features. They may contain some important information that might be missed by other powerful feature extraction techniques. The hybrid feature spaces can complement each other to enhance the prediction performance of a predictor.

### 3.3. Performance Comparison of Ensemble Learning Method and Individual Base Classifiers

In order to verify the strength of the proposed ensemble method, prediction results of JPPRED and its individual base classifiers, including RF, NB, LR, and RBF network, are investigated and compared. As presented in Table 2, compared with the 4 individual classifiers, JPPRED achieves slightly lower sensitivity for type III J-protein prediction. However, JPPRED has definite advantages in predicting the other 3 types of J-proteins. JPPRED yields sensitivity of 0.905 for type I J-proteins, 0.745 for type II J-proteins, and 1 for type IV J-proteins, about 4.8%, 5.4%, and 30% higher than that of the highest performing individual classifier, respectively. The AvgSn reflects the average discriminative power for different types of J-proteins. JPPRED achieves a satisfactory AvgSn of 0.875, about 35.1%, 8.9%, 36.4%, and 62.5% higher than that of 4 individual classifiers, respectively. In addition, JPPRED obtains balanced sensitivity and specificity for each type of J-proteins. On the contrary, individual base classifiers lead to high sensitivity and low specificity for type III J-proteins, low sensitivity, and high specificity for type I, II, and IV J-proteins. JPPRED obtains lower accuracy of 0.852 compared to that of RF, NB, and LBF metwork, which may be due to the imbalanced data. For the classification of imbalanced data, accuracy is not an appropriate measure because it may be still high when the sensitivity is very low [65]. These results indicate that combining different individual classifiers trained by balanced subdatasets can effectively enhance the prediction performance for predicting types of J-proteins and deal with the imbalanced data problem.

### 3.4. Feature Selection Results

**Figure 7:**
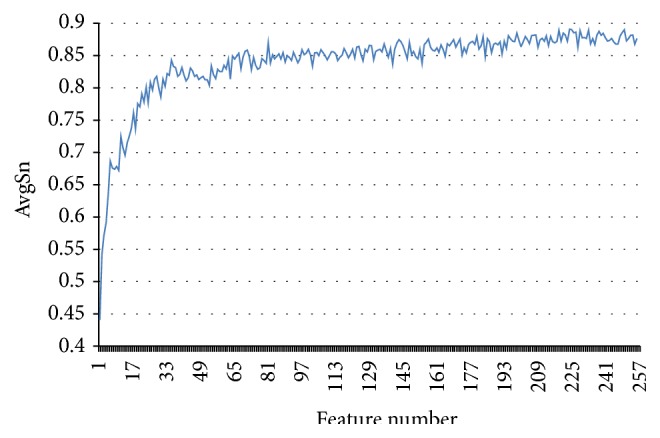
IFS curve that shows the values of AvgSn against feature subsets generated by the SU-IFS method.

SU lists the ranked 256 features with the maximum relevance to the class of samples. Then, the IFS method combined with ensemble learning method is employed to extract the optimal feature set. In the IFS procedures, adding the ranked features one by one from the SU list, 256 individual predictors are built for the corresponding 256 subfeature sets. We then test the prediction performance for each predictor and obtain the IFS results (see Table  S2).  Figure 7 gives the IFS curve with AvgSn as the *y*-axis and the number of features as the *x*-axis. The curve reaches its peak with the AvgSn of 0.891, when the first 224 features in the SU feature list are used. These 224 features are deemed as the optimal feature set. The predictive Acc based on these 224 features are 0.862.

### 3.5. Contribution of Feature Selection to the Ensemble Learning Method

We investigate the influence of feature selection on the performance of JPPRED. The prediction performance of JPPRED using feature selection or not by 10-fold cross validation is shown in Table 3. From Table 3, sensitivity and specificity for each type of J-proteins with feature selection are all significantly better than those without feature selection. Using feature selection, the number of features is reduced from 256 to 224 and the AvgSn, Acc are improved from 0.875 to 0.891 and 0.852 to 0.862, respectively. These results indicate that some noise is present in the original feature set due to the existence of redundant or uninformative features. SU-IFS can significantly reduce this noise to effectively improve the performance of JPPRED.

### 3.6. Analysis of Optimal Features

**Figure 8:**
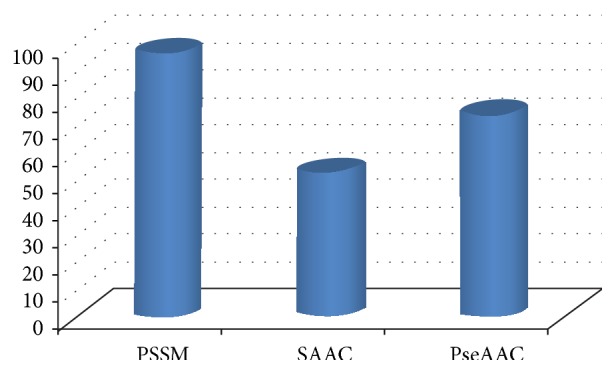
Distribution of each type of features in the optimal feature set.

The distribution of the number of each type of features in the optimal feature set is investigated and shown in Figure 8. From Figure 8, among the 224 optimal features, there are 97 PSSM based features, 53 SAAC based features, and 74 PseAAC based features, indicating that all types of features play some roles in the determination of types of J-proteins.

The percentage of the optimal features accounting for the corresponding feature types is also investigated. 97% of PSSM based features, 88.3% of SAAC based features, and 77.1% of PseAAC based features are chosen as the optimal features, indicating that PSSM based features play an irreplaceable role in predicting types of J-proteins. These results are in accordance with previous studies that evolution conservations exist in the J-domains or J-like domains and the frequencies of 20 nature amino acids are remarkably different among different types of J-proteins. The discriminative power of PseAAC based features is smaller compared to that of PSSM and SAAC. Though PseAAC based features rank low in the optimal feature list, they play a complementary role in improving the prediction performance of JPPRED.

### 3.7. Comparison with Existing Method

To further evaluate the effectiveness of the proposed method, it is essential to compare the performance of the present model with the previous predictors. The performance comparison results are shown in Table 4. Results in Table 4 show that [14] achieves high sensitivity of 0.986 for type III J-proteins, notably accompanied with extremely low sensitivities for type I, II, and IV J-proteins, respectively. Specificities of [14] present the opposite case. These results indicate that [14] cannot effectively deal with the imbalance between majority class and minority classes. On the contrary, JPPRED obtains balanced sensitivity and specificity for each type of J-proteins. The AvgSn of 0.891 using 224 features is significantly superior to that of [20] using 512 features. It is noted that because the number of type III J-proteins is extremely large, the samples in type III J-proteins tend to be identified correctly, which will lead to a large Acc value as given in [14]. Obviously, Acc is not a proper objective index for this serious data imbalance problem. We can draw the conclusion that JPPRED not only is indeed an effective and powerful approach for predicting the types of J-proteins but also can deal with the data imbalance problem. It is convinced that JPPRED will be a useful tool for J-proteins prediction.

From the results above, the excellent performance of JPPRED can be ascribed to 3 aspects. (1) JPPRED adopts multiple feature extraction strategies, including SAAC, PseAAC, and PSSM, which are related to the properties of different types of J-proteins. (2) JPPRED applies SU-IFS to select the high discriminative ones from original features, which can improve the prediction performance. (3) JPPRED proposes an ensemble classifier integrating multiple basic classifiers of diverse learning policies, which can not only overcome the drawbacks of individual classifiers but also deal with the serious class imbalance problem.

## 4. Conclusions

J-proteins, a prototypical molecular chaperone family, act as obligate cochaperone partners of the Hsp70 chaperone to participate in a variety of cellular processes. The distinct structures of peptide-binding sites for 4 types of J-proteins lead to remarkable differences in their chaperone functions and in the development of diseases. Therefore, accurate identification of types of J-proteins will be of benefit to reveal the mechanism of J-proteins and contribute to developing drugs to cure or alleviate diseases. In this study, an ensemble predictor called JPPRED has been presented with hybrid features extracted from SAAC, PseAAC, and PSSM. To solve the dimension disaster and improve the performance, SU-IFS method is adopted to obtain the optimal feature set. To deal with the data imbalance problem, the ensemble method is developed by integrating the 20 subclassifiers trained by 20 subdatasets. The average sensitivities of JPPRED based on 3 individual feature spaces are 0.791, 0.851, and 0.744, respectively, indicating the satisfying discriminative power of these features. PSSM based features discriminate the types of J-proteins with best performance among the 3 individual feature spaces. JPPRED yields the highest average sensitivity of 0.875 using the hybrid feature spaces of SAAC, PseAAC, and PSSM, indicating that the hybrid feature spaces can complement each other to enhance the prediction performance of a predictor. In addition, SU-IFS can significantly improve the performance of JPPRED with features reducing from 256 to 224. Analysis of optimal features reveals that all types of features play roles in the determination of types of J-proteins. To evaluate the prediction performance objectively, JPPRED is compared with previous study. JPPRED obtains balanced performance for each type of J-proteins with average sensitivity of 0.891, which is significantly superior to that of previous method. Therefore, JPPRED can be a potential candidate for predicting the types of J-proteins.

## Supplementary Material

Table S1. The benchmark dataset.
Table S2. The Incremental Feature Selection (IFS) result.

## Figures and Tables

**Table 1:** Performance of JPPRED using various feature spaces by 10-fold cross validation.

Feature space	AvgSn	Acc
SAAC	0.791	0.746
PSSM	0.851	0.808
PseAAC	0.744	0.606
SAAC + PseAAC	0.814	0.766
SAAC + PSSM	0.832	0.847
PseAAC + PSSM	0.863	0.821
SAAC + PseAAC + PSSM	0.875	0.852

**Table 2:** Performance of ensemble learning method and individual base classifiers by 10-fold cross validation.

Subfamily	Measure	Ensemble	RF	NB	LR	RBF network
Type I J-protein	Sn	0.905	0.698	0.857	0.556	0
	Sp	0.849	0.935	0.882	0.853	0.934

Type II J-protein	Sn	0.745	0.418	0.691	0.400	0
	Sp	0.857	0.947	0.890	0.858	0.927

Type III J-protein	Sn	0.851	0.979	0.895	0.889	1
	Sp	0.855	0.486	0.768	0.442	0

Type IV J-protein	Sn	1	0	0.700	0.200	0
	Sp	0.849	0.938	0.884	0.848	0.900

AvgSn		0.875	0.524	0.786	0.511	0.250

Acc		0.852	0.922	0.881	0.837	0.885

**Table 3:** Performance of JPPRED with or without feature selection by 10-fold cross validation.

Subfamily	Measure	No feature selection	Feature selection
Type I J-protein	Sn	0.905	0.921
	Sp	0.849	0.859

Type II J-protein	Sn	0.745	0.782
	Sp	0.857	0.866

Type III J-protein	Sn	0.851	0.861
	Sp	0.855	0.877

Type IV J-protein	Sn	1	1
	Sp	0.849	0.860

Dimension		256	224

AvgSn		0.875	0.891

Acc		0.852	0.862

**Table 4:** Performance comparison of the proposed method with [14].

Reference	Subfamily	Sn	Sp	Dimension	AvgSn	Acc
[14]	Type I J-protein	0.746	0.988	512	0.651	0.9406
	Type II J-protein	0.491	0.991			
	Type III J-protein	0.986	0.620			
	Type IV J-protein	0.381	1			

This study	Type I J-protein	0.921	0.859	224	0.891	0.862
	Type II J-protein	0.782	0.866			
	Type III J-protein	0.861	0.877			
	Type IV J-protein	1	0.860			
